# Asrij Maintains the Stem Cell Niche and Controls Differentiation during Drosophila Lymph Gland Hematopoiesis

**DOI:** 10.1371/journal.pone.0027667

**Published:** 2011-11-14

**Authors:** Vani Kulkarni, Rohan J. Khadilkar, Srivathsa M. S., Maneesha S. Inamdar

**Affiliations:** Molecular Biology and Genetics Unit, Jawaharlal Nehru Centre for Advanced Scientific Research, Bangalore, India; Stockholm University, Sweden

## Abstract

Several signaling pathways control blood cell (hemocyte) development in the *Drosophila* lymph gland. Mechanisms that modulate and integrate these signals are poorly understood. Here we report that mutation in a conserved endocytic protein Asrij affects signal transmission and causes aberrant lymph gland hematopoiesis. Mammalian Asrij (Ociad1) is expressed in stem cells of the blood vascular system and is implicated in several cancers. We found that *Drosophila* Asrij is a pan-hemocyte marker and localizes to a subset of endocytic vesicles. Loss of *asrij* causes hyperproliferation of lymph gland lobes coupled with increased hemocyte differentiation, thereby depleting the pool of quiescent hemocyte precursors. This co-relates with fewer Col+ cells in the hematopoietic stem cell niche of *asrij* mutants. *Asrij* null mutants also show excess specification of crystal cells that express the RUNX factor Lozenge (Lz), a target of Notch signaling. *Asrij* mutant lymph glands show increased N in sorting endosomes suggesting aberrant trafficking. *In vitro* assays also show impaired traffic of fluorescent probes in *asrij* null hemocytes. Taken together our data suggest a role for Asrij in causing increased Notch signaling thereby affecting hemocyte differentiation. Thus, conserved endocytic functions may control blood cell progenitor quiescence and differentiation.

## Introduction

The conservation of mechanisms as well as ontogeny of blood development over the course of evolution is well established [1], [2], [3]. Signaling proteins and transcription factors required for mediating hematopoiesis are conserved between vertebrate and *Drosophila* hematopoiesis [4], [5]. While several signaling molecules, receptors and transcription factors have been identified, mechanisms required for transmittance of the signal are poorly understood. Endocytic proteins form part of the cellular trafficking machinery and are expected to play an integral role in modulating signals and their effectors. We therefore investigated the role of an identified hemocyte-expressed endocytic protein Asrij in *Drosophila* hematopoiesis.

We previously reported *asrij* expression in *Drosophila* hemocytes [6]. Asrij was first identified as a conserved protein expressed in embryonic stem (ES) cells and the developing blood vasculature [7] and is also a mouse hematopoietic stem cell marker [8]. Expression is initiated in the mouse mesoderm prior to and overlapping with that of the hemangioblast marker Flk1/VEGFRII, persists in the blood islands and continues in the developing vasculature [7]. Similarly early onset of *asrij* expression is also seen in *Drosophila* prohemocytes and is independent of the prohemocyte marker Serpent (Srp) [6]. Asrij protein has a novel OCIA domain with two conserved helices and named after the human ortholog Ovarian Carcinoma Immunoreactive Antigen domain 1 (Ociad1). Mouse Asrij localizes to endocytic vesicles [7]. A *Drosophila* yeast two hybrid screen [9] reported that Asrij interacts with ADP ribosylation factor 1 (ARF1) a GTPase that functions in endocytosis and recycling. The mutant phenotype of *asrij*/*ociad1* has not been reported. However, mis-regulation of *ociad1* is associated with several hematological neoplasms [10], [11] such as multiple myeloma [12] and neutrophilia [13]. To elucidate the conserved functions of *asrij* in hematopoiesis, we undertook a functional analysis of Asrij in *Drosophila*.

Drosophila lymph gland is the best studied site of hematopoiesis. Lymph gland hemocytes are released only at metamorphosis [14], [15], [16] or prematurely upon immune challenge [17], [18], [19]. At the end of embryogenesis the lymph gland exists as a single paired primary lobe anterior to the cardiac tube [20]. The primary lobe of the third instar larval lymph gland is demarcated into immature and mature hemocyte zones [4], [5], [21]. The outer cortical zone (CZ) houses mature hemocytes of the myeloid lineage comprised of P1- expressing plasmatocytes and Lz- expressing crystal cells which, in the larva, are released into circulation only upon immune challenge [21]. In addition a specialized hemocyte, the lamellocyte, is induced in response to parasitic wasp infection and is marked by the L1 antigen [22]. The inner medullary zone (MZ) is comprised of pro-hemocytes which express Domeless-GAL4 and Drosophila E-Cadherin (DE-cad) [21]. A subset of Antennapedia (Antp)- expressing cells in the primary lobe forms the posterior signaling centre (PSC) which is the hematopoietic stem cell niche [23]. The JAK-STAT, Wingless (Wg) and Hedgehog (Hh) pathways [23], [24], [25] generate multiple signals that act in the PSC and medullary zone and are integrated to control stem cell maintenance, precursor quiescence and lineage differentiation.

By the third instar the lymph gland has additional secondary and tertiary lobes residing between segments T3 to A3 [4]. The origin of these additional lobes is widely debated but poorly understood [21]. They are thought to contain undifferentiated prohemocytes [15]. The lymph gland lobes and nephrocytic pericardial cells (PCs) [26] flank the cardiac tube and show a characteristic arrangement and spacing along the anterior-posterior axis [27].

In this report, we use genetic analyses to reveal an important role for endocytic proteins in hematopoiesis. We show that Asrij is expressed in embryonic and lymph gland hemocytes. A null mutation in *asrij* leads to a dramatic increase in the number of lymph gland lobes. Asrij blocks hemocyte precursor differentiation and controls hemocyte number. We present a detailed analysis of the hematopoietic defects associated with *asrij* mutants. We also show that Asrij modulates Notch signaling and discuss the importance of endosomal trafficking in hematopoiesis. These results provide definitive genetic evidence that loss of *asrij* promotes aberrant cell proliferation and differentiation *in vivo* and will help enhance our understanding of pathways affected in hematopoietic disorders.

## Materials and Methods

### Fly stocks and genetics

*Drosophila* stocks were maintained under standard rearing conditions at 25°C. Canton-S was used as the wild type reference strain. Respective UAS or GAL4 parent stocks or w1118 were used as controls where appropriate. P element stock KG08017 (Bloomington # 14935) was used to generate excision lines of *asrij* by following standard procedure (see Text S1 and Figure S2). For expression in transgenic flies, *asrij* cDNA (BDGP clone ID AT12418) was cloned in pPUAST vector [28]. The construct was injected according to standard procedures [29]. Germline transformed, transgenic flies were selected by red eye color (w+) and maintained as homozygotes. Multiple transgenic lines were analyzed for each construct. For knock down experiments, UAS-*Dmasrij-*RNAi transgenic flies were obtained from National Institute of Genetics, Japan. Other stocks used in this study were *HemolectinGAL4* (Bloomington # 6395), *e33cGAL4* (K.Anderson, NY) and *lzGAL4UASGFP* (Bloomington # 6314), *domelessGal4;UAS-mCD8GFP* (M. Crozatier, Toulouse, France).

### Immunostaining and microscopy

Immunostaining was performed on embryos as described previously [30]. Wandering third instar larvae were used for dissection of lymph glands. All dissections were in phosphate-buffered saline (PBS). Dissected preparations were fixed in 4% formaldehyde in PBS for 30 min then transferred to tubes. All subsequent steps were with gentle agitation on a flat bed rotator, using 1 ml of each solution at room temperature, except for the antibody incubations, which were at 4°C. Hemolymph was extracted into 150 µl of Schneider's complete medium (CM; Schneider's insect medium supplemented with 10% FBS (GIBCO), 1 µg/ml bovine pancreatic insulin, 150 µg/ml penicillin, 250 µg/ml streptomycin, 750 µg/ml glutamine) by puncturing the larval integument using fine forceps. Hemocytes were allowed to attach for one hour, fixed with 2.5% paraformaldehyde, permeabilized with 0.4% Igepal for 13 min, pre- incubated in blocking solution (BS; medium with 2 mg/ml BSA) and followed by incubation with primary antiserum diluted in BS. Excess antiserum was washed off and cells were incubated with labeled secondary antibodies diluted in BS. Images were captured with a Zeiss LSM510-Meta confocal microscope and analyzed using LSM510 processing software (Carl Zeiss, Inc.). Rabbit polyclonal antibodies were raised against the full-length recombinant Asrij protein expressed in *E*. *coli*. Antisera were checked for specificity to the immunogen by Western blot analysis (see Text S1 and Figure S1). Other antibodies were against: Serpent (1∶800) [31], Pvr (1∶1000) [32], Rab5 (1∶50) [33], Rab11 (1∶1000) [34], dArl8 (1∶500) [35], GM130 (1∶500) [36], Hrs (1∶1000) [37], Collier (1∶50) [38], Antenapedia (1∶20, Developmental Studies Hybridoma Bank, # 4C3), NICD (1∶50, Developmental Studies Hybridoma Bank, # C17.9C6), Odd (1∶400) [39], Phospho histone H3 (Upstate # 09-797), and mAbs H2, P1, C4 and L1 (1∶50) [40]. Secondary antibodies were Alexa-488 or Alexa-568 conjugated (Molecular Probes, Inc.).

### Molecular biology

Total RNA was extracted from embryo, larvae, pupae and adults using Trizol reagent (Invitrogen Bioservices). cDNA was prepared using Superscript enzyme (Invitrogen Bioservices) and used as a template for PCR amplification. qRT-PCR was performed using SYBR green chemistry in a Rotor Gene 3000 (Corbett Life Science 3000) and analyzed with the accompanying software. Primer sequences used for RT-PCR and qRT-PCR are provided in Table S1.

### Hemocyte counts

Circulating hemocyte counts were obtained as described before [41] from wandering third instar larvae. Hemocyte counts were expressed as per animal equivalent. Appropriate control genotypes were included to take care of variation due to genetic background.

## Results

### Asrij is a pan-hemocyte marker

**Figure 1:**
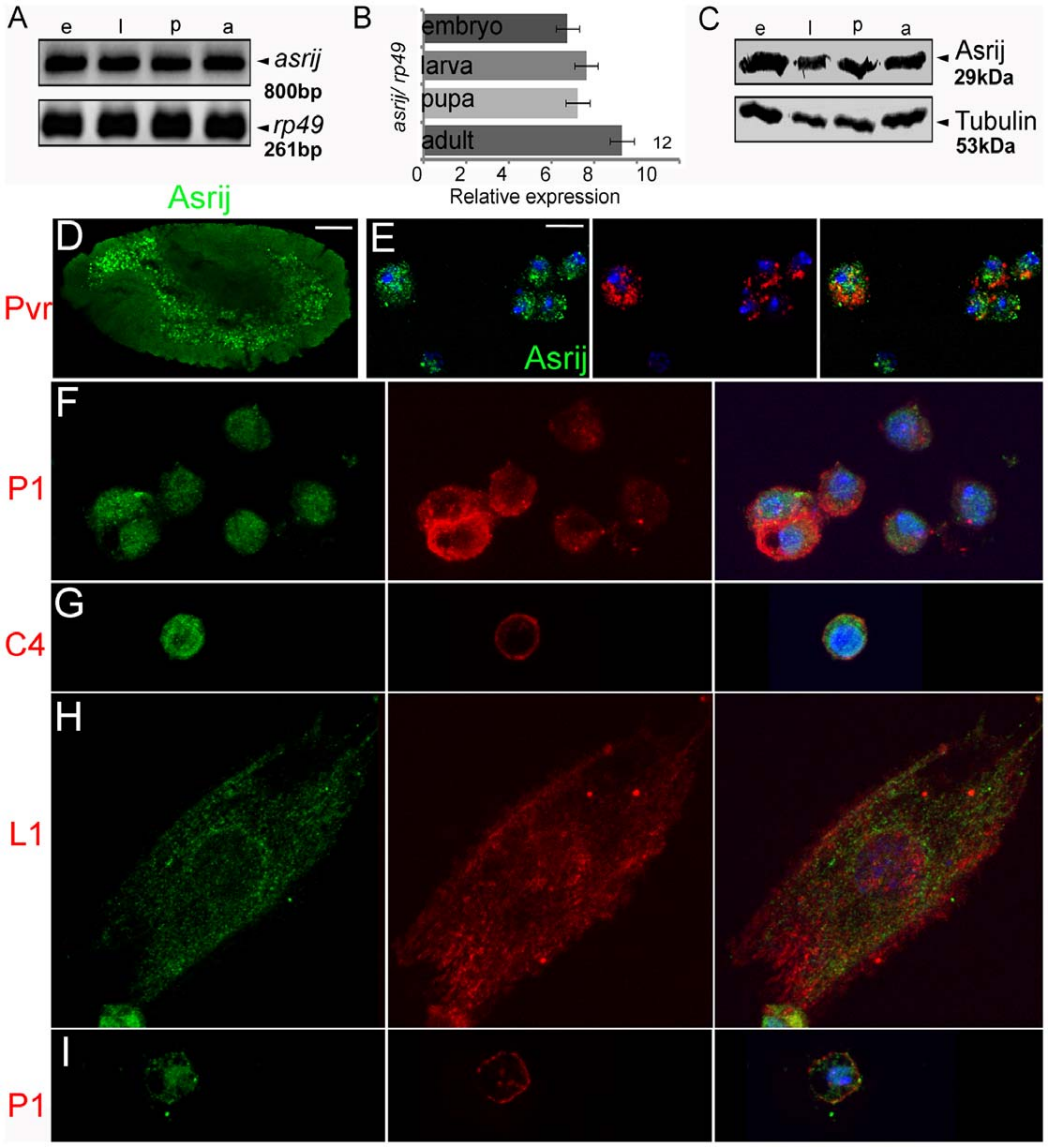
Asrij is expressed at all stages of development and is a pan hemocyte marker. (A–C) Estimation of (A, B) *asrij* transcript levels by RT-PCR (A) and quantitative RT-PCR (B) and (C) Asrij protein levels by Western blot analysis at different developmental stages. e: embryo, l: larva, p: pupa and a: adult. *rp49* and Tubulin levels were used to estimate RNA and protein loading respectively. (D–I) Asrij expression (green) in embryonic (D, E), larval (F–H) and adult (I) hemocytes. (D, E) Expression in the whole embryo (D) and in isolated embryonic hemocytes co-stained for Pvr (red) (E). (F–H) Expression in larval hemolymph hemocytes co-immunostained (red) to identify P1 expressing plasmatocytes (F), C4 expressing crystal cells (G) and L1 expressing lamellocytes (H). (I) Adult circulating hemocytes co-stained for P1 (red). (E–I) Nuclei are stained with DAPI (blue). Scale bar: (D) 50 µm (E–I) 5 µm.

**Figure 2:**
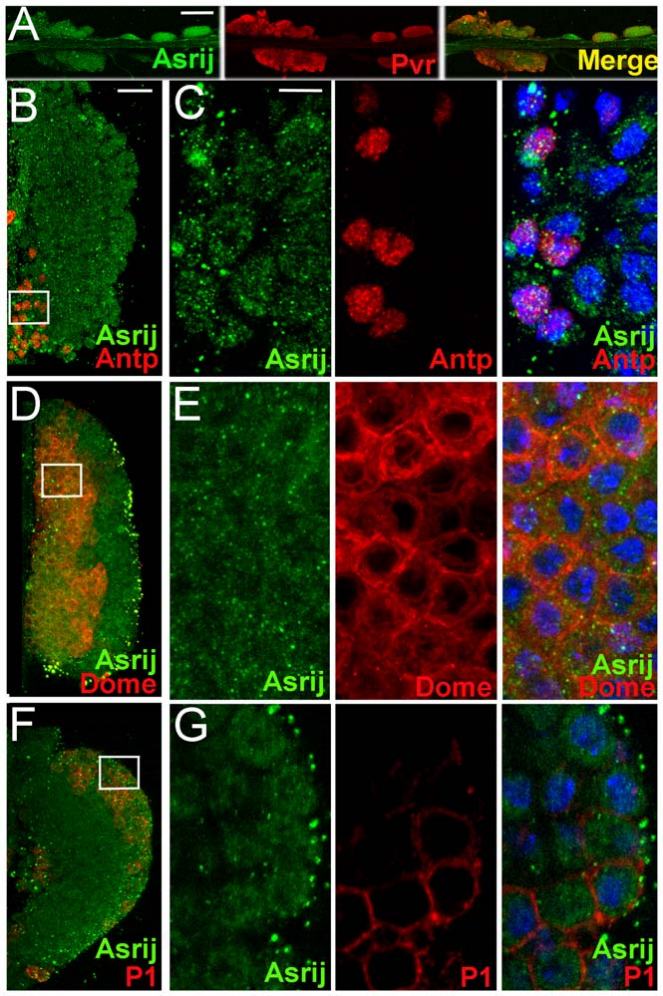
Asrij is expressed in all cells of the larval lymph gland. Third instar larval lymph gland immunostained to show expression of Asrij (green) and various lymph gland markers (red) as indicated. (A) Whole lymph gland showing Asrij (green) expression in primary and secondary lobes co-immunostained with Pvr (red). (B–G) Primary lymph gland lobe showing expression of Asrij (green) along with (B, C) the posterior signaling center marker Antennapedia, (D, E) medullary zone marker *domelessgal4UASmCD8GFP* stained with anti-GFP and (F, G) cortical zone marker P1 respectively. (C, E, G) Corresponding high magnification images of boxed region in (B, D, F) respectively. Nuclei are stained with DAPI (blue). Scale bar: (A) 50 µm (B, D, F) 20 µm (C, E, G) 5 µm.

Earlier we reported *asrij* mRNA expression in embryonic hemocytes [6]. Here we undertook a detailed expression analysis of *asrij* RNA by Reverse Transcription-Polymerase Chain Reaction (RT-PCR) and of protein by Western blot analysis and immunolocalization at different developmental stages of *Drosophila melanogaster*. We analyzed *asrij* mRNA expression at the whole animal level and found that it is present at all developmental stages (Figure 1A) and relative levels are comparable as seen by quantitative RT-PCR (Figure 1B). Polyclonal antibodies against the full- length protein (Text S1 and Figure S1) revealed an approximately 29 kDa protein expressed throughout development (Figure 1C). Immunolocalization showed the protein was present in embryonic hemocytes (Figure 1D, 1E). Asrij is also expressed in all subsets of larval (Figure 1F–H) and adult (Figure 1I) hemolymph hemocytes such as P1^+^ plasmatocytes, C4^+^ crystal cells and L1^+^ lamellocytes. In addition, we saw Asrij expression in the larval lymph gland lobes (Figure 2A). To identify cell types in the primary lobe that express Asrij we co-stained for Asrij and PSC, MZ or CZ markers Antp, domeless (using a GFP reporter) and P1 respectively. Asrij is expressed in all cells of the primary lymph gland lobe (Figure 2B–G). Asrij expression could not be detected in several other tissues examined (Figure S1). Specificity of the Asrij antibody was confirmed by using pre-immune serum and no primary antibody controls (not shown) as well as by staining the null mutant hemocytes (Figure S3). Thus, Asrij is a marker for all hemocyte lineages during development and would aid in further studies on hemocyte development and function.

### Drosophila Asrij is an endocytic protein

Mouse Asrij localizes to the endocytic vesicles in endothelial cells [7]. Comparison of the vertebrate and *Drosophila* Asrij sequences showed a 42% conservation which is primarily in the OCIA domain (Figure S2A). To check whether this conservation is reflected in the protein localization we used antibodies raised against *Drosophila* Asrij to see its localization in hemocytes. Asrij showed punctate staining decorating the cytoplasm of hemocytes to varying extents (Figures 1 and 2). Co-immunostaining for several sub-cellular markers showed that Asrij is present in Rab5^+^ early endosomes, Rab11^+^ recycling endosomes, dArl8^+^ lysosomes and GM130^+^ Golgi compartment (Figure 2B–E). These data suggest that Asrij could play a role at various steps of endocytosis.

### Loss of asrij promotes hyperproliferation of secondary lymph gland lobes

The larval lymph gland is a good model for studying conserved mechanisms in definitive hematopoiesis [4]. Hence, we analyzed *asrij* function in lymph gland hematopoiesis. For this we generated an *asrij* null mutant (*arj^9^/arj^9^* homozygous recessive) (Figure S3A–F) as well as flies bearing *asrij* knockdown and over-expression constructs under the control of the UAS-GAL4 system (Text S1). The null mutant is viable, fertile and with no apparent developmental defect, though it lacks *asrij* RNA (Figure S3B) and protein (Figure S3D–F). *asrij* knockdown cells had greatly reduced Asrij expression (≤50%) in all hemocyte classes (data not shown).

**Figure 3:**
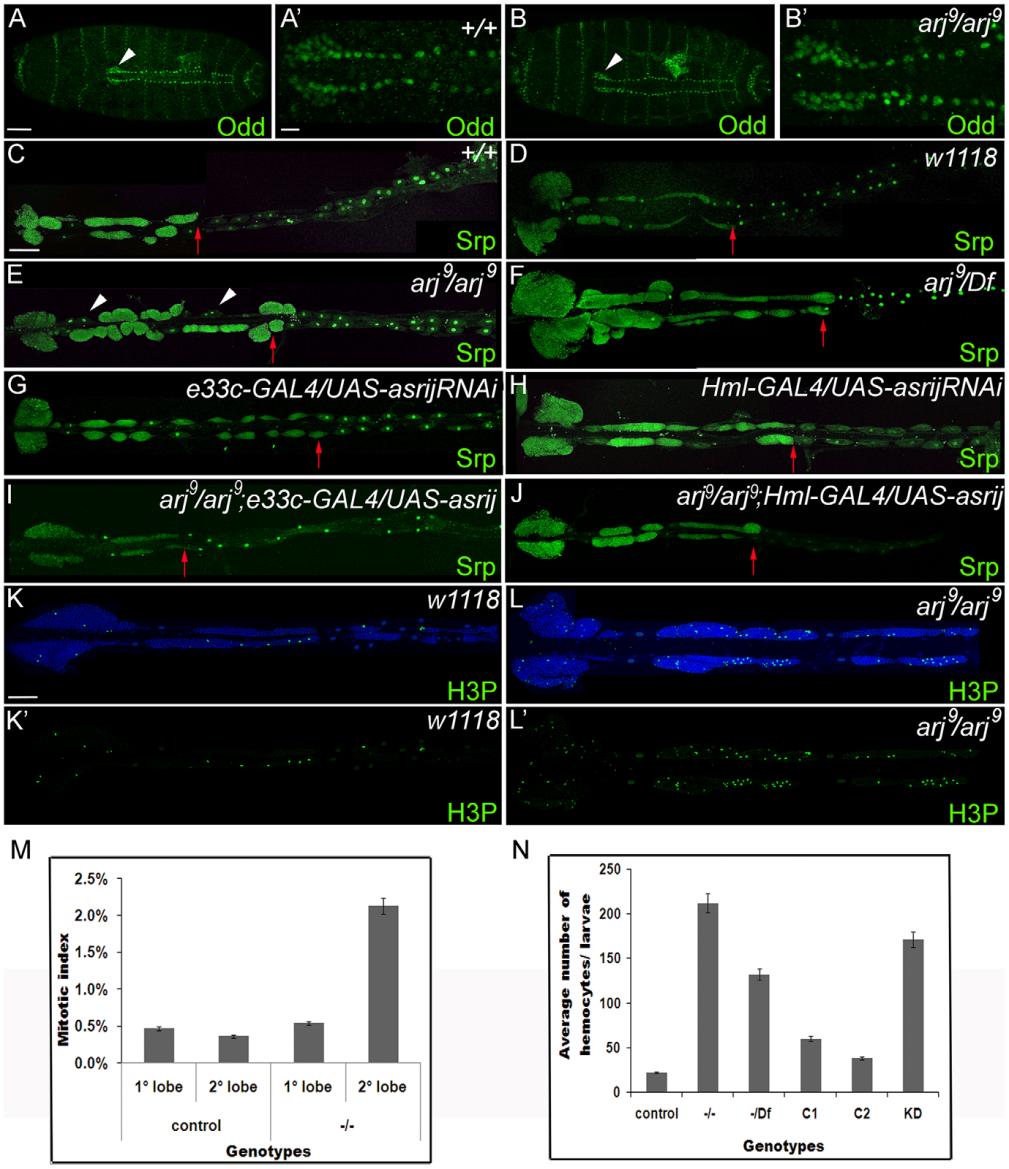
Asrij mutants show excess of lymph gland lobes. (A–B') Embryonic lymph gland of wild type control (+/+) and *asrij* mutant (*arj^9^/arj^9^*) stained to show expression of Odd (green). (A', B') Magnified view of the region marked by arrowheads in (A) and (B). (C–J) Dorsal vessel stained for expression of Serpent (green) in various genotypes as indicated. Red arrow indicates the posterior most lymph gland lobe seen. (C, D) are wild type and control genotypes respectively. (E) *asrij* null mutant homozygotes (F) *asrij* null mutant/Df, (G, H) *asrij RNAi* knockdown (I, J) *asrij* null mutant phenotype rescued by *asrij* over-expression. (K–L') H3P staining (green) on third instar larval lymph gland of control (K, K') and *arj^9^/arj^9^* (L, L'). (K,L) also show nuclei stained with DAPI (blue); (M) Graphical representation of mitotic index (H3P+ cells/total cells) in primary and secondary lobes of control and *asrij* null mutant (−/−) larvae, n = 10. (N) Average hemocyte number/larva of control, (−/−) *asrij* null mutant, (−/*Df*) *asrij* null mutant/Df, C1 (e33cGal4 parent), C2 (*UASasrij* parent) and KD (*e33cGal4/UASasrij RNAi*). Scale bar: (A, B) 20 µm (A', B') 10 µm (C–J) 100 µm (K–L') 50 µm.

The primary lymph gland lobes are specified in the late embryo and can be identified by the expression of Srp and Odd-skipped (Odd). Staining for Odd expression showed that *arj^9^*/*arj^9^* embryonic lymph glands (Figure 3B, 3B') were comparable in size and cell number to that of wild type (Figure 3A, 3A'). 2–4 pairs of secondary lobes containing undifferentiated hemocyte precursors arise in the late second instar and grow significantly large by the third instar (Figure 3C). All *asrij* null mutant larvae (*arj^9^/arj^9^*) showed higher number of secondary lymph gland lobes which expanded into posterior segments up to A4 or A5 (Figure 3E) as compared to controls (Figure 3D). The mutant ectopic lobes were asymmetric and extended up to three quarters of the dorsal vessel length. *arj9/Df* larvae showed a similar phenotype (Figure 3F). This phenotype was also seen in over 60% of larvae where *asrij* expression was knocked down by RNA interference using different GAL4 drivers (Figure 3G–H) (n>50). Two transgenic RNAi lines generated with different constructs showed a similar phenotype. Excess secondary lobes were not seen in the *asrij* mutant embryos (Figure 3B) or second instar larvae (not shown) indicating that they arose late in larval life. Though pericardial cell number was not significantly altered in *asrij* mutants, the arrangement was drastically affected (Figure 3E). Further, over-expression of Asrij in lymph glands of the null mutant with either of the GAL4 drivers restored lymph gland lobe number and pericardial cell arrangement to a near wild type pattern (Figure 3I–J).

We also examined mitosis in the lymph gland by staining for histone H3 phosphorylated at serine 10 (H3P), which is an indicator of mitosis. There was no significant change in the number of mitotic foci in the primary lobe of *asrij* null mutant as compared to wild type. However the mutant secondary lobes showed a dramatic increase in mitotic foci (Figure 3K–M), indicating greatly increased cell proliferation.

We next examined the hemocyte number in circulation. The total number of circulating hemocytes was significantly increased in *asrij* mutants (*arj^9^/arj^9^*; *arj^9^/Df* and *asrij*-knockdown) as compared to controls (Figure 3N). This suggests *asrij* controls hemocyte number by regulating hemocyte proliferation and/or differentiation.

### Premature loss of medullary zone in asrij mutants

**Figure 4:**
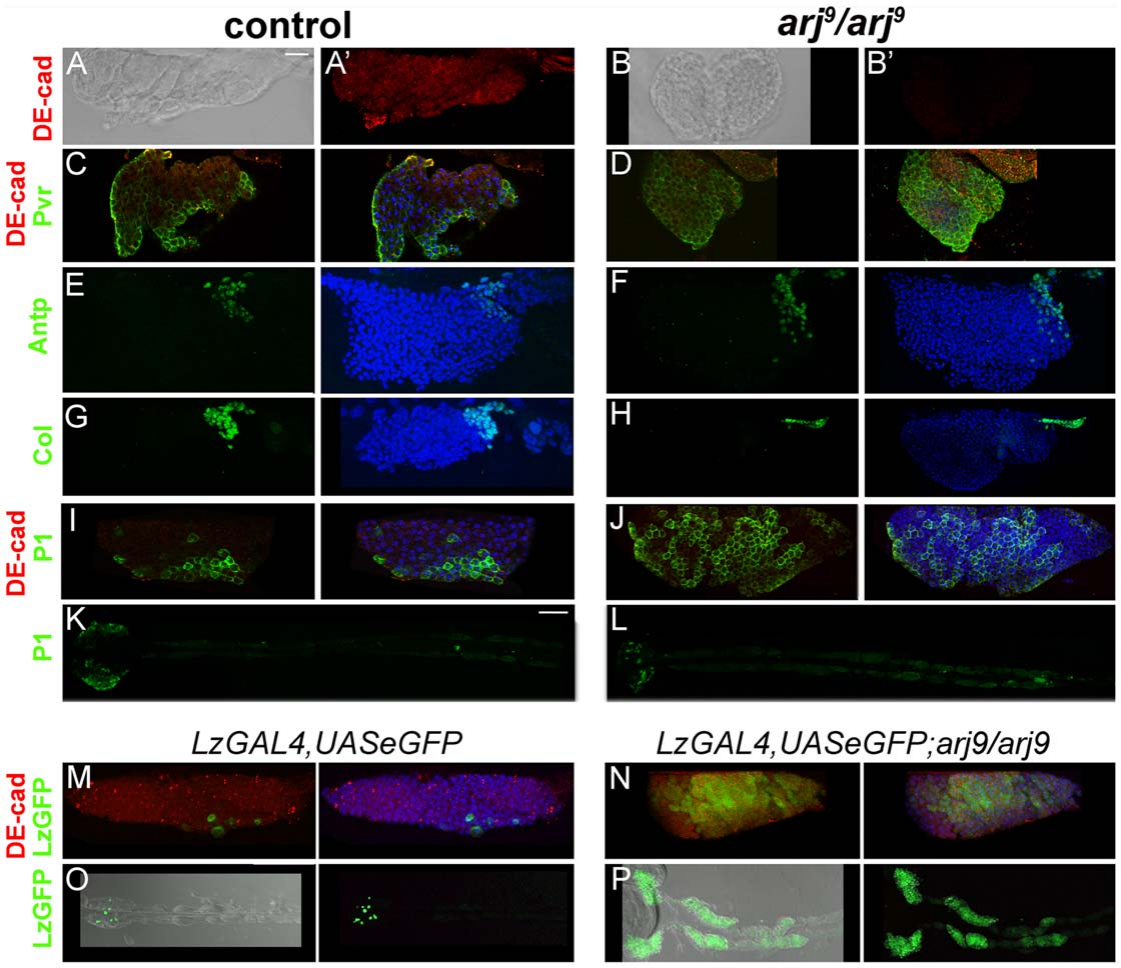
Premature loss of medullary zone and hemocyte differentiation in *asrij* mutants. (A–P) Confocal microscopy images of control [*w1118* or LzGAL4/UASeGFP] and *arj^9^/arj^9^* larval lymph gland as indicated. (A–J) and (M–N) show only the primary lobe. (A, B) Phase contrast images showing loose packing of lymph gland cells in *asrij* mutant (B) as compared to *w1118* control (A) which shows tightly packed cells in the MZ. (A', B') show DE-cadherin expression (red). (C–J) Expression (green) of (C–D) Pvr, (E–F) Antp, (G–H) Col, (I–J) P1 and (M–N) Lz. (A–D, I–J and M–N) also show expression of the MZ marker DE-cad (red). Zone of DE-cad expression is reduced in *asrij* mutant (B') as compared to wild type (A'). Note that the *asrij* mutant lobe has fewer Col^+^ cells (H), increased P1^+^ plasmatocytes (J, L) and increased Lz^+^ crystal cells (identified by GFP) (N, P) as compared to respective controls. No P1^+^ cells are detected in secondary lobes of control and *arj^9^/arj^9^.* (O–P) Increased Lz-GFP expression is seen in secondary lobes of *LzGAL4,UASeGFP; arj^9^/arj^9^* (P) as compared to *LzGAL4,UASeGFP* (O) lymph glands. (C–J, M and N) Nuclei stained with DAPI (blue). Scale bar: (A–J, M–N) 20 µm (K–L, O–P) 100 µm.

One characteristic feature of the lymph gland is the segregation of cell types based on their stage of maturation and differentiation [21] which can be correlated with gene expression. The peripheral CZ has loosely packed cells while the MZ is compact [21]. We examined whether loss of *asrij* affects this organization. In the *asrij* mutant lobe (Figure 4B), cells were loosely packed compared to control MZ (Figure 4A). This co-related with altered gene expression. The number of DE-cadherin expressing cells was greatly reduced and staining was diffuse in the mutant lobe (Figure 4B'). Consequently, an apparent increase in the zone of Pvr^+^ cells was seen (Figure 4D compare to 4C). Since the MZ is at the inner core of the lobe, confocal sections at the centre were always carefully analyzed separately to avoid counting peripheral CZ cells. These data suggest that the expanded CZ is due to premature differentiation of MZ cells. Hence, *asrij* could affect the maintenance and fate of hemocyte precursors in the lymph gland.

The lymph gland MZ is specified and maintained by signals from the PSC. The PSC is specified in the embryo by Antp- and Collier (Col) - expressing cells. The primary role of the PSC in the third instar larva is to act as a hematopoietic stem cell niche whereby it signals to maintain hemocyte precursors in the medullary zone. During metamorphosis or upon immune challenge the precursors differentiate [21]. Lack of or reduction in Col^+^ cells in the PSC causes medullary zone cells to differentiate prematurely [25]. We examined expression of Antp and Col in the PSC of *asrij* mutants. There was no appreciable change in the number of Antp^+^ cells indicating normal PSC specification in *asrij* null mutants (32.2±6.2 in mutant as compared to 32.4±7.8 in controls, n = 5). (Figure 4F compare to 4E). However a significant reduction in Col^+^ cells in the mutant (20±5.7) as compared to control (38.4±11.2) suggested a role for *asrij* in maintenance of Col^+^ cells (Figure 4H compare to 4G). Alternatively, the specified Col^+^ precursors may not be able to divide in the absence of Asrij. However, *asrij* mutant lymph glands showed increased labeling for H3P (see Figure 3L) suggesting that proliferation may be normal. These data suggest that PSC maintenance is compromised in *asrij* mutants.

### Premature differentiation of hemocyte precursors in asrij mutant lymph gland

Plasmatocytes and crystal cells are the two major hemocyte types present in the CZ. The P1 antigen marks differentiated plasmatocytes in the CZ [21] (Figure 4I). In *asrij* mutant a large number of P1^+^ plasmatocytes were seen all over the primary lobe (Figure 4J). Interestingly, no P1^+^ cells were seen in the secondary lobes of control and mutant lymph glands (Figure 4K–L). Lz is a marker of precursor and differentiated crystal cells [3], [42]. We examined Lz expression in lymph glands using the *lz-Gal4,UAS-GFP* enhancer trap line to drive GFP expression (Figure 4M–P). Numerous GFP^+^ cells were seen all over the primary lobe and also in secondary lobes of *asrij* mutant lymph glands (Figure 4N, 4P) indicating premature differentiation of hemocytes.

These data suggest that maintenance of precursor populations depends on *asrij* expression and may be regulated by signaling pathways that operate in lymph gland development.

### Asrij mutants show aberrant Notch trafficking

**Figure 5:**
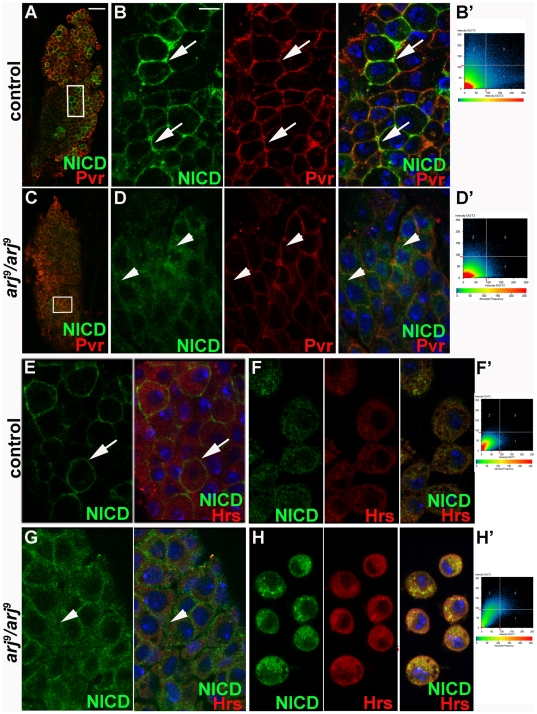
Notch Intracellular Domain (NICD) is entrapped in Hrs^+^ endosomes. NICD expression (green) in larval primary lymph gland lobe (A–E, G) or circulating hemocytes (F, H) of control (A–B, E–F) and *asrij* null mutant (C–D, G–H) genotypes as indicated. Lymph gland cells were also co-immunostained for expression of the hemocyte membrane marker Pvr (red) (A–D) or the endosomal marker Hrs (red) (E–H). (B, D) are high magnification images of the boxed area in (A, C) respectively. (B', D', F' and H') show co-localized pixels of a single confocal section from (B, D, F, H) respectively. Arrows indicate cells with membrane localized NICD and arrowheads indicate cytoplasmic, diffuse localization of NICD. Note the significant co-localization between NICD and Pvr in control (B') and between NICD and Hrs in mutant (H'). In each set, panels to the extreme right are merged images. Control and the *asrij* null mutants were always imaged at the same settings. Nuclei are stained with DAPI (blue). Scale bar: (A, C) 20 µm. (B, D, E–J) 5 µm.

During larval hematopoiesis Serrate (Ser)-mediated signaling through the Notch (N) pathway, results in specification of Lz^+^ crystal cells [3], [4], [43]. Lz is expressed in crystal cell precursors and in mature crystal cells in the cortical zone of the primary lymph gland lobe and to a lesser extent in a small population of circulating hemocytes. Since N function is mandatory for larval crystal cell differentiation [44], we next examined whether there was any effect on N signaling. Mutant lymph glands showed a significant change in staining pattern for the intracellular domain of Notch (NICD) (compare diffuse staining in Figure 5C, 5D to membrane localized staining in Figure 5A, 5B) which could reflect in altered target gene expression. Re-localization of NICD suggested that it may have a positive effect on N signaling (see below). Increased N signal should result in a greater Lz expression and hence increased crystal cell specification as we had seen (Figure 4N). This suggests that *asrij* could potentially restrict the domain of Lz expression by affecting N signaling.

Interestingly, *asrij* mutant lymph gland cells showed a change in NICD localization. Co-staining for the membrane-localized lymph gland marker Pvr showed that while NICD was localized primarily to the membrane in control cells, *asrij* mutant cells showed diffuse NICD staining (Figure 5D compare to Figure 5B). This indicates aberrant localization of NICD in *asrij* mutants. During N signal transduction NICD is cleaved by γ-secretase and transported to the nucleus through a series of endocytic compartments [45], [46], [47]. Aberrant NICD localization in mutant cells suggests that Asrij could regulate N trafficking. To analyze the effect of Asrij on N signaling further, we examined the expression of N and various trafficking proteins in the *asrij* mutant lymph gland hemocytes compared to controls (Figure 5E, 5G). For a higher resolution analysis of this phenotype we checked expression in control and *arj^9^*/*arj^9^* hemolymph hemocytes (Figure 5F, 5H). Immunostaining showed NICD trapped in sub-cellular compartments of *arj^9^*/*arj^9^* hemocytes, which we identified as Hrs^+^ endosomes (Figure 5G, 5H). Hrs is required for maturation of endosomes into multivesicular bodies (MVBs) [48], [49]. Control hemocytes showed very little co-localization of NICD with Hrs (Figure 5F'), whereas there was increased overlap between the two in *asrij* mutant hemocytes (Figure 5H'). To analyze the ability of *asrij* null hemocytes to traffic generic molecules, we used fluorescent probes. FITC-labelled dextran (F-dex) is used as an indicator of molecular movement within the cell during endocytosis [50]. Trafficking of F-dex in hemocytes has been well-documented [50]. *Asrij* null hemocytes showed greatly reduced uptake of the probe (Figure S4).

Based on these results we reasoned that in *asrij* null hemocytes N is stalled in the endosomes due to lack of Asrij. Therefore Asrij is required for NICD trafficking. Notably, we did not see any effect on NICD localization in tissues where *asrij* is not normally expressed, such as the wing disc.

## Discussion

We have used *Drosophila* hematopoiesis as a model to study the role of a conserved endocytic molecule in trafficking of signals required for maintenance of stem cells and precursors. Mutants lacking the endocytic protein have excess hemocytes in circulation, hyperproliferation of lymph gland secondary lobes and premature differentiation of hemocytes. In agreement with our previous reports on mouse and *Drosophila asrij* we have shown that Asrij is expressed from the earliest stages of prohemocyte specification. While embryos homozygous for a deficiency of *asrij* (and therefore deleted in multiple genes) are lethal (Inamdar 2003), *asrij* null mutant is homozygous viable. Just as mutations in human *asrij* are associated with cancers [51], *Drosophila asrij* loss-of-function mutants also cause hyperproliferation and premature differentiation of precursors, indicating that the mutant phenotype is the result of perturbation in conserved gene function. Our results indicate that Asrij interacts with multiple signaling pathways and will be an important tool in the analysis of hematopoiesis.

### Asrij regulates lymph gland proliferation

A remarkable feature of the *arj^9^* mutant phenotype is the supernumerary posterior lymph gland lobes. The origin of the secondary lymph gland lobes is not understood and no precursors are detected in the embryo [21]. In *asrij* mutants we see a bona fide primary lobe and excess secondary lobes arising in the larva. This suggests the presence of previously undetected larval lymph gland precursors whose proliferation was suppressed by Asrij. Alternatively, *asrij* may suppress specification of posterior lymph gland progenitors in larval development. In addition, *asrij* may control proliferation of circulating hemocytes as we see increase in their number. Similar phenotypes were reported for other mutants that show overgrowth in mutant lymph glands and increase in circulating hemocytes [41]. *Asrij* mutants provide an excellent tool to elucidate events in hematopoiesis and interrogate signaling pathways implicated in proliferation of lymph gland lobes.

The intriguing question remains of how Asrij can promote both proliferation and differentiation in posterior lobes and differentiation in the primary lobe. Multiple signals in the anterior lobe are integrated in time and space to maintain the PSC and control precursor differentiation. These may provide mechanisms independent of or complementary to Asrij function in the control of proliferation. Such details are not available for the secondary lobes, which are believed to harbor a homogenous population of quiescent precursors. Loss of Asrij leads to hyperplastic effects in the secondary lobes. Hyperplasia is also a phenotype associated with excess N signaling. We propose that *asrij* controls proliferation by integrating with Notch signaling. Previous studies report the effect of N signaling on the primary lobe or circulating hemocytes [44], but the secondary lobes have not been analyzed in detail. Loss of *asrij* leads to increased Notch signaling and hence hyperproliferation in secondary lobes. However, additional signals required to maintain quiescence may be absent and hence there is increased differentiation to Lz^+^ crystal cells, which is a read out for Notch signaling. On the other hand, plasmatocyte differentiation is not seen in *asrij* mutant secondary lobes. This suggests that pathways that promote plasmatocyte differentiation are not active in the posterior or not controlled by Asrij.

### Non-autonomous or secondary effects of Asrij

As none of the hemocyte-specific GAL4 drivers is reported to function only in the lymph gland primary and secondary lobes, we used the best available drivers e33cGAL4 (expressed in all lymph gland cells and in other tissues) [52] and HmlGAL4 (expressed only in the primary lobe and in hemocytes) [53] to generate *asrij* knockdown or overexpression flies. Experiments using either GAL4 driver gave similar phenotypes to those using the null mutant (*arj^9^/arj^9^*) or the *arj^9^/Df* flies. These results validate that the phenotypes seen are primarily due to the effect on *asrij*. Though HmlGal4 is reported to drive expression only in the primary lobe and hemocytes, multiple experiments that we have done clearly show that expression using HmlGAL4 affects function in the secondary lymph gland lobes. This could be either due to previously unreported low level or leaky activity of the driver in posterior lobes or due to a non-autonomous effect of manipulating *asrij* activity in primary lobes and in circulating hemocytes. The interaction between hemocytes in lymph gland posterior lobes and in hemolymph merits further investigation.

As Asrij is involved in vesicular traffic, it may affect multiple signaling pathways and possibly have non-autonomous or secondary effects. Though *asrij* mutants show disturbed pericardial cell arrangement, the heartbeat of *arj^9^* homozygous larvae is normal (data not shown) indicating no functional effect on cardiac rhythm. This is in agreement with earlier reports that pericardial cells are not required for normal cardiac function [26].

### Asrij is required for maintenance of the stem cell niche and precursor quiescence

Inspite of reduced Col^+^ cells in *asrij* mutant, no appreciable reduction in Antp^+^ cell number was seen, suggesting that Asrij may affect maintenance of Col^+^ cells, which needs to be investigated. The reduced Col^+^ PSC in *asrij* mutants could affect MZ quiescence. Cells in the MZ are compact, bounded by extracellular matrix (ECM) and maintained in a slow-cycling quiescent state by signals from the PSC [5]. Loss of MZ accompanied by increased differentiation and release of hemocytes into circulation is normally seen during metamorphosis or upon immune challenge. For this, precursor- matrix interactions have to be modulated as cells differentiate and migrate to the periphery where they are loosely packed [21]. The choice between prohemocyte maintenance and its differentiation may be mediated by changes in ECM components and in adhesive properties of a cell. *Asrij* null lymph glands have loosely packed cells with greatly reduced expression of the Wg target DE-cad (Figure 4B,B'). The importance of deregulated adhesion in cancer is well documented. Inactivation of E-cadherin in human and mouse is associated with progression to metastasis and also promotes neoplasia. Increased proliferation of precursors in the *asrij* mutant lymph gland and increase in circulating hemocyte number suggest that *asrij* may act on mechanisms that control DE-cad expression and indirectly control cell adhesion. Ociad1 plays a key role in human cancer cell adhesion [51]. Changes in Ociad1 expression levels can modulate integrin function thereby affecting cell adhesion and the ability of cancer cells to form secondary colonies [54]. We speculate that Asrij/Ociad1 may play a similar role in regulating adhesion via the Wnt pathway. Further, this change in adhesive properties could influence the choice between stem cell maintenance and differentiation.

### Control of endocytosis is important for hematopoiesis

Premature hemocyte differentiation in *asrij* mutant larvae suggests a regulatory role for endocytosis during normal development. Control levels of Asrij are required to prevent hemocyte differentiation possibly as a secondary effect of MZ loss. Alternatively, *asrij* may attenuate signals required for hemocyte differentiation through uptake and degradation of signaling molecules. In the absence of Asrij, control on signal amplitude may be lost and can result in initiation of the differentiation program. Retention of NICD in subcellular compartments correlates well with increased Lz^+^ cells and supports the latter. Further, this phenotype does correlate with a N gain of function as seen by increase in crystal cells. Vaccari et al. [55] reported NICD entrapped in Hrs positive endosomes in ESCRT mutants – (in genes like tsg101, vps25, vps 20)- showed Notch gain of function phenotypes such as overgrowth of eye imaginal discs and eye phenotypes in adult mutant flies too. The mechanism by which *asrij* affects NICD endocytosis merits further investigation.

Asrij may have context-dependent functions during hematopoiesis. Our observations that *asrij* mutants show increased N signaling reveals a mechanism by which endocytic molecules can regulate cell proliferation. Further, NICD is aberrantly localized in all mutant lymph gland cells compared to only a subset of control lymph gland cells. This is reflected in the widespread ectopic Lz^+^ cells in mutants compared to controls. This suggests Asrij interacts with additional pathways that control N signaling.

Hemocytes also differentiate and are released into circulation during systemic infection. One possibility that remains to be tested is whether Asrij is a target of the signaling cascade triggered by immune challenge. Reduction in Asrij levels could help rapidly respond to immune challenge and we are testing whether this is so. This scenario is also supported by the presence of excess circulating hemocytes in the *asrij* mutant. Expression of *asrij* only in the lymph glands is sufficient for complete rescue of the mutant phenotype, indicating a function for Asrij within the lymph gland and hemocytes. However the signaling molecules regulated by *asrij*, or their effectors could be released from the lymph gland or fat body to activate systemic targets in the larva. Further studies on the Asrij protein and its role in cellular traffic would help address these mechanisms.

### Common endocytic pathways could mediate signaling during hematopoiesis

In addition to interaction of Asrij with ARF1 [9], the human ortholog Ociad1 is predicted to interact with SLC35F2 a solute carrier family protein and also with KDR, the human Flk1/VEGFRII homolog (http://string.embl.de/newstring_cgi/show_network_section.pl). Hematopoiesis in *Drosophila* is governed by a transcription factor cascade initiated by the GATA factor Srp. Hemocyte division, density and possibly viability are controlled by the Toll/Cactus and JAK/STAT pathways which also activate immunity genes [17], [56]. Similar phenotypes of Asrij and the conservation in expression pattern and functions suggest common endocytic pathways that mediate hematopoiesis. Asrij being an endocytic protein could be involved in regulation of these multiple inputs. Human ociad1 interacts with several cellular proteins [54], supporting our hypothesis.

Notch processing is quite complex and several tissue- specific components are implicated in its activation [57], [58]. Notch accumulates in intracellular structures when endocytic progression is perturbed resulting in its hyperactivation leading to hyperplasia [37], [59], [60], [61]. We have shown that loss of Asrij leads to retention of Notch intracellular domain in Hrs endosomes correlating with increased Notch activity, seen as increase in Lz^+^ cells. Up-regulation of Notch has been implicated in human blood cell disorders such as, T cell acute lymphoblastic leukemia [62]. Mutants such as *lethal giant discs* (*lgd*) that affect protein sorting in the late endosomes or MVB result in Notch gain- of- function phenotypes [63]. Our preliminary analysis with fluorescent probes indicates a generic requirement for Asrij in intracellular transport in hemocytes. Further investigation is required to understand whether Notch activation in *asrij* mutant is ligand dependent or independent. *Asrij* mutants provide an excellent tool to understand the mechanism involved in precocious N signaling leading to blood cell disorders. Further *asrij* mutants are viable and can be used in studying post-embryonic Notch signaling in various contexts.

### Asrij functions at multiple levels during hematopoiesis

The importance of signaling proteins, receptors and transcriptional targets of the N and Wg pathways for cancer development is well established. In contrast, data regarding endocytic molecules that traffic the pathway components and modulate their activity is limited. Here we show that loss of the endocytic protein Asrij affects *Drosophila* at multiple levels leading to increased hematopoiesis by enhancing precursor proliferation and differentiation. Our results indicate a role for Asrij in PSC maintenance, which in turn affects precursor quiescence. In addition, a more direct role for Asrij is implicated in crystal cell specification via control of NICD traffic.

Our study demonstrates the value of a comparative approach in identifying functions of conserved mammalian genes in *Drosophila*. The early onset of Asrij expression during development suggests that it could be a key player in vertebrate hematopoiesis as well. By virtue of its ability to control cellular traffic, Asrij may control cell adhesion, proliferation and differentiation, which makes it difficult to tease out the exact mechanism of its action. Understanding how *asrij* controls the balance between stem cell number and committed precursors may aid in disease correction and regenerative medicine. Together, our findings indicate that endocytosis is a key modulator of lymph gland hematopoiesis and provide *in vivo* demonstration that genetic loss of endocytic components can lead to accelerated hematopoietic development and facilitate premature differentiation.

## Supporting Information

Figure S1 (**A–B**) **Antigen-antibody competition assay to validate the specificity of Asrij antibody.** Western blot showing specificity of Asrij (A) antibody. Lanes: (1, 2) Blot probed with antibody preincubated with 25 or 50 µg of corresponding antigen. (3, 4) blot probed with antibody without preincubation with antigen. (B–C) Asrij expression (green) by immunostaining with anti-Asrij antibodies could not be detected in several other tissues examined including wing disc (B), fat body (C). Nuclei stained with DAPI (blue). Panels to the extreme right are merged images. Scale bar: (B, C) 50 µm.
(TIF)

Figure S2 **Conservation and subcellular localisation of Asrij in** ***Drosophila melanogaster*** **.** (**A**) Schematic representing conservation in OCIA domain of Asrij. The N half of the Asrij protein including predicted helices are conserved in *Drosophila*, mouse and human. (**B–E**) Subcellular localization of Asrij. Immufluorescence analysis of hemocytes stained for expression of Asrij (green, extreme left panels) and subcellular marker proteins (red, middle panels) such as (B) Rab5, (C) Rab11, (D) dArl8 and (E) GM130. Nuclei are stained with DAPI (blue). Panels to the extreme right in each set are merged images. Scale bar: (B–E): 5 µm.
(TIF)

Figure S3 **Southern blot analysis confirms insertion in** ***arj^9^*** **mutant.** (A) Schematic showing the details of the *asrij* null mutant. (B) Southern blot of Hind III digested genomic DNA from *asrij* excision lines probed with ^32^P-labelled cDNA. Lanes. 1: CS, 2: BL14935, 3: *arj* ^9^/*arj* ^9^ and 4: Marker. A 3.2 kbp band of expected size is seen in wild type whereas *arj* ^9^/*arj* ^9^ mutant has 2 bands of 2.4 kbp and 1.3 kbp due to 550 bp remnant of P element sequence. Analysis of *asrij* (**C**) transcript expression by RT-PCR and (**D**) protein expression by immunoblot with anti-Asrij antibody. Genotypes are as indicated above the lanes. (**E–F**) Immunofluorescence analysis of Asrij (green) expression in hemocytes of wild type (E) and *arj^9^*/*arj^9^* mutant (F). Hemocytes are identified by the expression of the pan hemocyte marker Hemese (red). Nuclei marked by DAPI (blue). Scale bar: (E, F) 5 µm.
(TIF)

Figure S4 **Dextran uptake is reduced in Asrij null hemocytes.** (**A**) Total cell associated fluorescence of internalized FITC Dextran 5 min after starting the incubation of wild type (CS), *asrij* null (*arj^9^/arj^9^*) and rescue (*arj^9^/arj^9;^* HmlGAL4/UAS Dm*asrij*) hemocytes (P = 0.002). (**B–C**) Representative images of wild type (B) and *arj^9^*/*arj^9^* mutant (C) hemocytes showing the uptake of FITC Dextran. Cell boundary is marked by a white line. Scale bar: (B, C) 5 µm.
(TIF)

Table S1 List of primers used for RT-PCR and qRT-PCR.
(DOC)

Text S1 (DOC)
