# Correction: Asrij Maintains the Stem Cell Niche and Controls Differentiation during Drosophila Lymph Gland Hematopoiesis

**DOI:** 10.1371/annotation/bed03b6b-8dfa-4aef-882c-b7e37bc122a4

**Published:** 2012-04-03

**Authors:** Vani Kulkarni, Rohan J. Khadilkar, Srivathsa S. Magadi, Maneesha S. Inamdar

There was an error in the third author's name. Srivathsa S. Magadi is correct. The correct citation is: Kulkarni V, Khadilkar RJ, Magadi SS, Inamdar MS (2011) Asrij Maintains the Stem Cell Niche and Controls Differentiation during Drosophila Lymph Gland Hematopoiesis. PLoS ONE 6(11): e27667. doi:10.1371/journal.pone.0027667 

