# Supplementary figures and images for: Asrij Maintains the Stem Cell Niche and Controls Differentiation during Drosophila Lymph Gland Hematopoiesis
Source: PLoS One. 2011 Nov 14;6(11):e27667. doi: 10.1371/journal.pone.0027667 (PMC3215734; doi:10.1371/journal.pone.0027667)

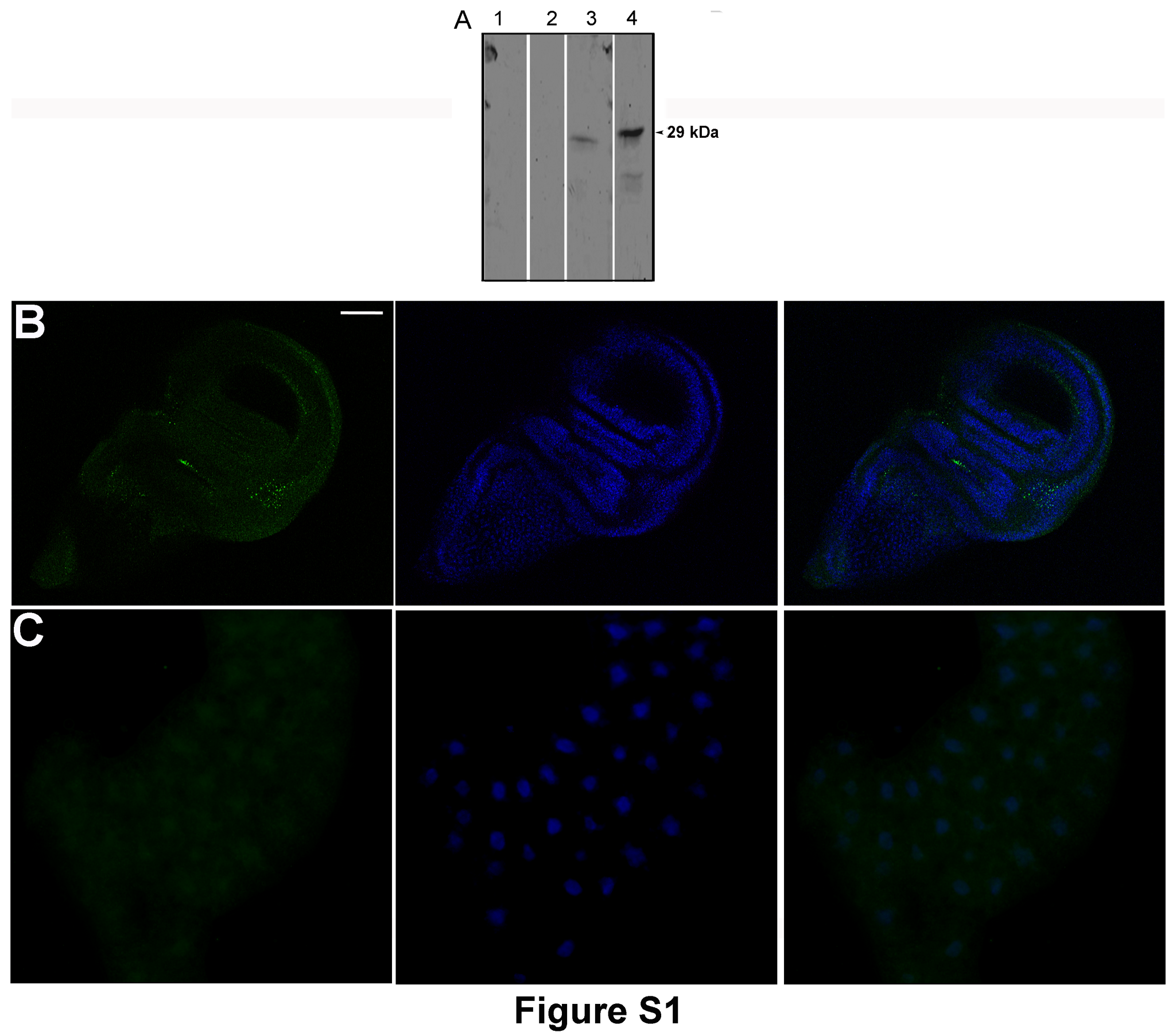

Supplement: Figure S1 — (A–B) Antigen-antibody competition assay to validate the specificity of Asrij antibody. Western blot showing specificity of Asrij (A) antibody. Lanes: (1, 2) Blot probed with antibody preincubated with 25 or 50 µg of corresponding antigen. (3, 4) blot probed with antibody without preincubation with antigen. (B–C) Asrij expression (green) by immunostaining with anti-Asrij antibodies could not be detected in several other tissues examined including wing disc (B), fat body (C). Nuclei stained with DAPI (blue). Panels to the extreme right are merged images. Scale bar: (B, C) 50 µm. (TIF) [file pone.0027667.s001.tif]

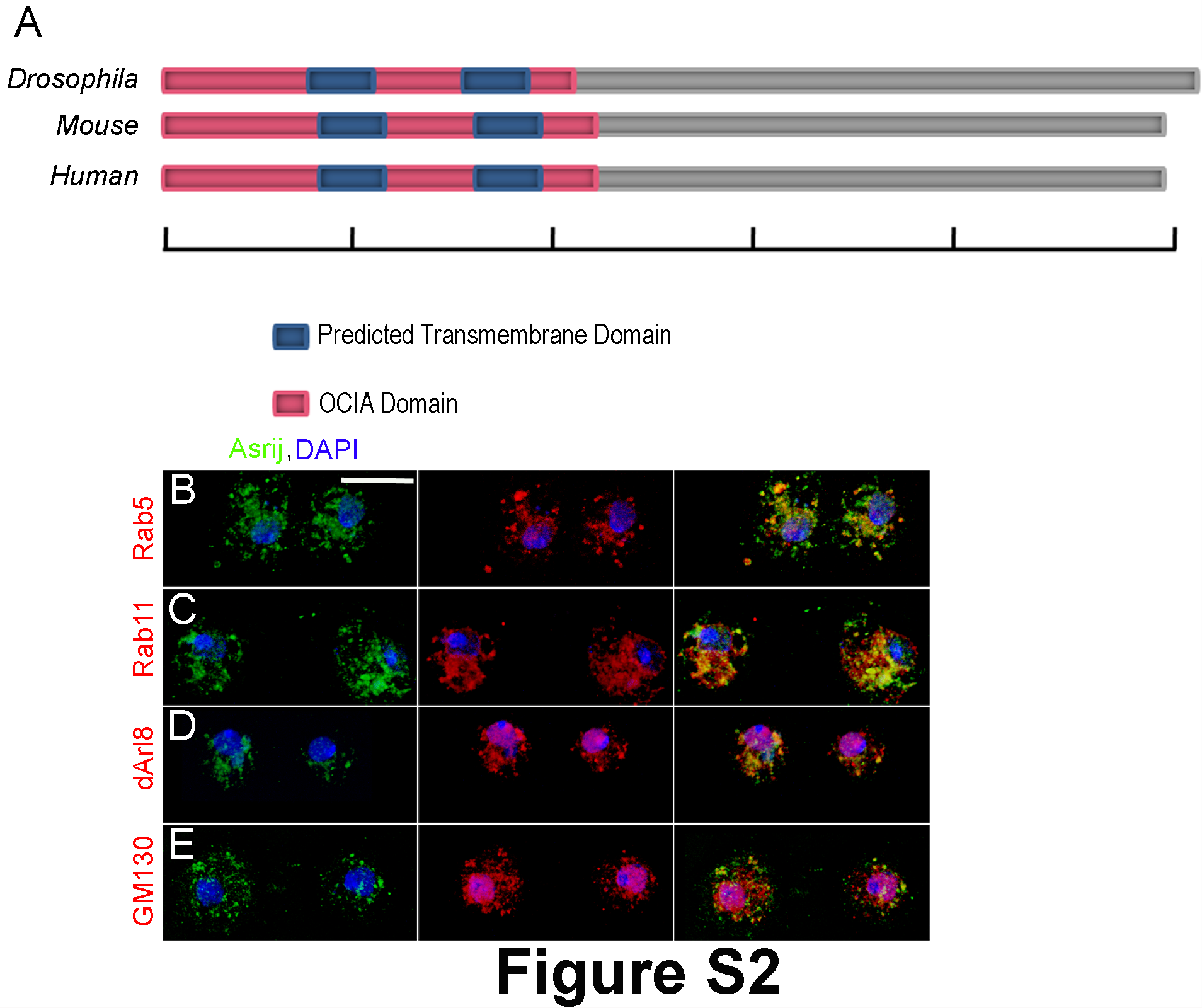

Supplement: Figure S2 — Conservation and subcellular localisation of Asrij in Drosophila melanogaster . (A) Schematic representing conservation in OCIA domain of Asrij. The N half of the Asrij protein including predicted helices are conserved in Drosophila, mouse and human. (B–E) Subcellular localization of Asrij. Immufluorescence analysis of hemocytes stained for expression of Asrij (green, extreme left panels) and subcellular marker proteins (red, middle panels) such as (B) Rab5, (C) Rab11, (D) dArl8 and (E) GM130. Nuclei are stained with DAPI (blue). Panels to the extreme right in each set are merged images. Scale bar: (B–E): 5 µm. (TIF) [file pone.0027667.s002.tif]

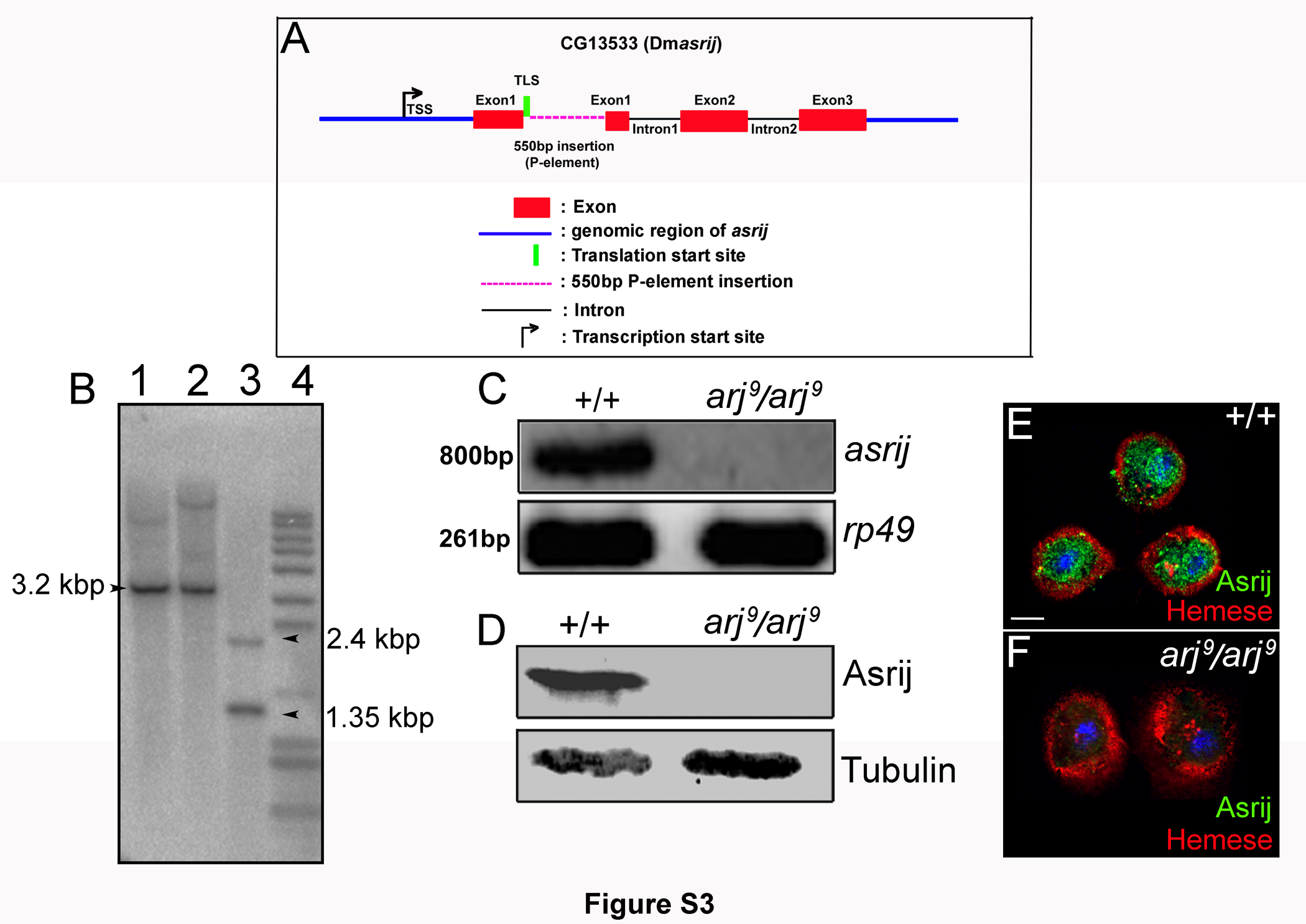

Supplement: Figure S3 — Southern blot analysis confirms insertion in arj9 mutant. (A) Schematic showing the details of the asrij null mutant. (B) Southern blot of Hind III digested genomic DNA from asrij excision lines probed with 32P-labelled cDNA. Lanes. 1: CS, 2: BL14935, 3: arj 9/arj 9 and 4: Marker. A 3.2 kbp band of expected size is seen in wild type whereas arj 9/arj 9 mutant has 2 bands of 2.4 kbp and 1.3 kbp due to 550 bp remnant of P element sequence. Analysis of asrij (C) transcript expression by RT-PCR and (D) protein expression by immunoblot with anti-Asrij antibody. Genotypes are as indicated above the lanes. (E–F) Immunofluorescence analysis of Asrij (green) expression in hemocytes of wild type (E) and arj9/arj9 mutant (F). Hemocytes are identified by the expression of the pan hemocyte marker Hemese (red). Nuclei marked by DAPI (blue). Scale bar: (E, F) 5 µm. (TIF) [file pone.0027667.s003.tif]

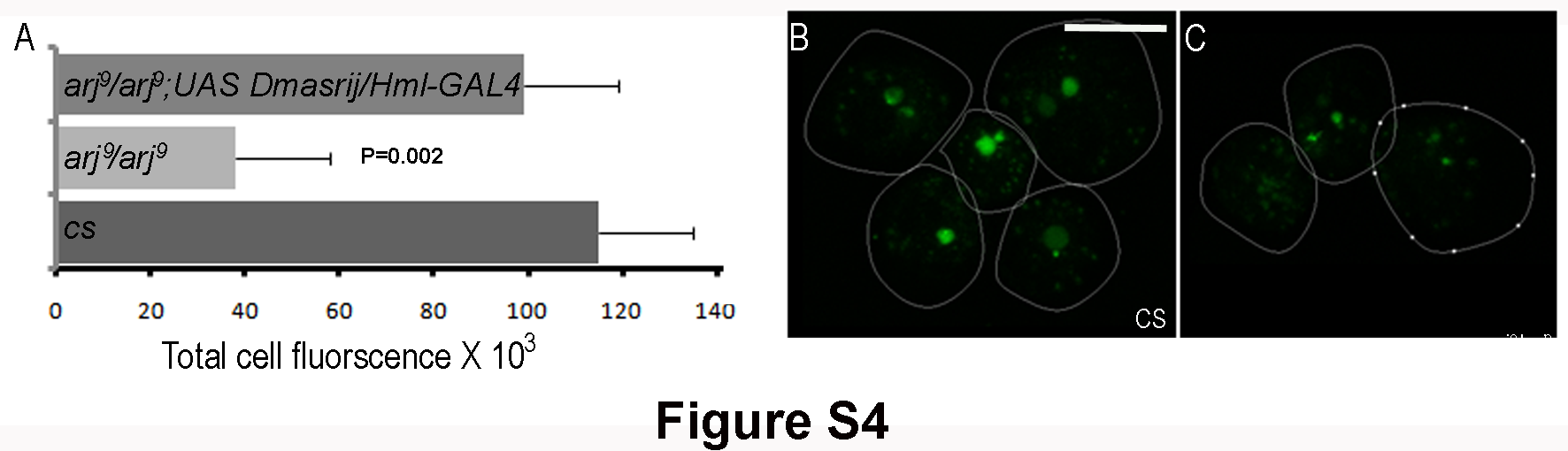

Supplement: Figure S4 — Dextran uptake is reduced in Asrij null hemocytes. (A) Total cell associated fluorescence of internalized FITC Dextran 5 min after starting the incubation of wild type (CS), asrij null (arj9/arj9) and rescue (arj9/arj9; HmlGAL4/UAS Dmasrij) hemocytes (P = 0.002). (B–C) Representative images of wild type (B) and arj9/arj9 mutant (C) hemocytes showing the uptake of FITC Dextran. Cell boundary is marked by a white line. Scale bar: (B, C) 5 µm. (TIF) [file pone.0027667.s004.tif]
