# Supplementary material for: Asrij Maintains the Stem Cell Niche and Controls Differentiation during Drosophila Lymph Gland Hematopoiesis
Source: PLoS One. 2011 Nov 14;6(11):e27667. doi: 10.1371/journal.pone.0027667 (PMC3215734; doi:10.1371/journal.pone.0027667)
Supplement: Table S1 — List of primers used for RT-PCR and qRT-PCR. (DOC) [file pone.0027667.s005.doc]

**Supplementary Tables**

**Table S1**. List of primers used for RT-PCR and qRT-PCR.

| Primer | Primer Sequence (5’-3’) |
| --- | --- |
| DmarjFL F | TGTATCACCATGGACTCCCCGCTGAACGATG |
| DmarjFL R | TTGTCGACTCGAGATCAGTCCAGGAGTCGCCG |
| Rp49 F | CCGCTTCAAGGGACAGTATC |
| Rp49 R | ACA ATC TCC TTG CGC TTC TTG |
