# Supplementary material for: Asrij Maintains the Stem Cell Niche and Controls Differentiation during Drosophila Lymph Gland Hematopoiesis
Source: PLoS One. 2011 Nov 14;6(11):e27667. doi: 10.1371/journal.pone.0027667 (PMC3215734; doi:10.1371/journal.pone.0027667)
Supplement: Text S1 — (DOC) [file pone.0027667.s006.doc]

**Supplementary information**

**Asrij Maintains the Stem Cell Niche and controls differentiation during Drosophila Lymph Gland Hematopoiesis.**

Vani Kulkarni, Rohan J. Khadilkar, Srivathsa M.S. and Maneesha S. Inamdar^1^

**Materials and methods**

**Generation of anti-Asrij antibody**. The *asrij* ORF (aa 1-257) was cloned into NcoI-XhoI sites of pET23d expression vector. The recombinant protein was expressed in *Escherichia coli* and purified by Ni–NTA binding followed by electroelution. Polyclonal antibodies were raised in rabbit and rat and were analysed by Western blot. Both antisera and pre-immune serum were treated with caprylic acid to purify IgG (Harlow and Lane, 1988). The specificity of antisera was checked by antigen-antibody competition analysis. 25 µg or 50 µg of recombinant protein was pre- incubated with the antibody and the blocked antibody was used to probe the Western blot containing antigen. The absence of bands in lanes 1 and 2 (Figure S1A) probed with blocked antibodies indicates specificity of the antibody for the immunogen. A 1:500 dilution of the caprylic acid- purified polyclonal antiserum was used for immunostaining.

**Generation of *asrij* mutants:** Using standard P element-mediated mutagenesis we generated several *Drosophila* lines in which a P element residing in the first exon of *asrij* was excised. One line (*arj^9^*) showed a 550 bp remnant of the P element (Supplementary data and Figure S3A, B) resulting in a protein null mutant, as confirmed by RT-PCR, Western blot analysis and immunostaining (Figure S3C-F). The insertion was confirmed by Southern blot (Figure S3B), PCR and sequencing analysis and the mutant did not complement a deficiency in the region. We also achieved knockdown of *asrij* expression by RNA interference using a *UASasrijRNAi* line and a lymph gland- and hemocyte- specific GAL4 driver *hemolectinGAL4* (*hml-GAL4*). Developmental time and morphology of *asrij* mutants is the same as that of Canton-S at 25 ^o^ C.

**Southern blot analysis**. Genomic DNA was extracted from *asrij* excision lines following the protocol described in Rubin and Spradling (Rubin and Spradling 1982). 40 flies were homogenized gently in 400 µl of homogenization buffer –B (0.2M Sucrose, 0.1M Tris, pH 9.2, 50mM EDTA and 0.5% SDS) on ice. The homogenate was incubated at 70° C for 30 minutes, and then 56 µl of 8M potassium acetate was added and incubated on ice for 30 minutes, followed by centrifugation for 15 minutes at 13000 rpm at 4°C. DNA from supernatant was precipitated with equal volume of Isopropanol, pelleted and then dissolved in TE buffer with RNase A (50 µg/ml). Hind III digested genomic DNA was Southern blotted and hybridized according to standard protocols as described in Maniatis (2003). The blot was probed with α -^32^P dCTP-labelled cDNA (Amersham) (Figure S3A).

**FITC dextran uptake assay:** FITC-labeled dextran (F-dex) uptake assays on hemocytes were performed as described before [25]. Images were analyzed using the software LSM Image Examiner (Carl Zeiss, Inc.). (Figure S4).

**Supplementary References**

1. Harlow, E. L., E. (1988). “Antibodies; A Laboratory Manual.” Cold Spring Harbor Laboratory, Cold Spring Harbor, NY.
2. J. Sambrook and D. Russell (2003). “Molecular Cloning: A Laboratory Manual.”
3. Rubin, G. M. and A. C. Spradling. 1982. Genetic transformation of Drosophila with transposable element vectors. Science 218:348-53.
